# Oropharyngeal Aspiration of *Burkholderia mallei* and *Burkholderia pseudomallei* in BALB/c Mice

**DOI:** 10.1371/journal.pone.0115066

**Published:** 2014-12-11

**Authors:** Kevin L. Schully, Matthew G. Bell, Jerrold M. Ward, Andrea M. Keane-Myers

**Affiliations:** 1 Naval Medical Research Center-Frederick, Frederick, Maryland, United States of America; 2 Global Vet Pathology, Montgomery Village, Maryland, United States of America; Public Health England, United Kingdom

## Abstract

*Burkholderia mallei* and *Burkholderia pseudomallei* are potentially lethal pathogens categorized as biothreat agents due, in part, to their ability to be disseminated via aerosol. There are no protective vaccines against these pathogens and treatment options are limited and cumbersome. Since disease severity is greatest when these agents are inhaled, efforts to develop pre- or post-exposure prophylaxis focus largely on inhalation models of infection. Here, we demonstrate a non-invasive and technically simple method for affecting the inhalational challenge of BALB/c mice with *B. pseudomallei* and B. *mallei*. In this model, two investigators utilized common laboratory tools such as forceps and a micropipette to conduct and characterize an effective and reproducible inhalational challenge of BALB/c mice with *B. mallei* and *B. pseudomallei*. Challenge by oropharyngeal aspiration resulted in acute disease. Additionally, 50% endpoints for *B. pseudomallei* K96243 and *B. mallei* ATCC 23344 were nearly identical to published aerosol challenge methods. Furthermore, the pathogens disseminated to all major organs typically targeted by these agents where they proliferated. The pro-inflammatory cytokine production in the proximal and peripheral fluids demonstrated a rapid and robust immune response comparable to previously described murine and human studies. These observations demonstrate that OA is a viable alternative to aerosol exposure.

## Introduction

The genus *Burkholderia* is comprised of numerous infectious species including *Burkholderia pseudomallei* and *Burkholderia mallei*. *B. pseudomallei*, a tropical soil saprophyte, is the etiological agent of melioidosis [1], [2]. The bacteria are sporadically endemic throughout the world between the 20th North and South Parallels but “hyper-endemic” in South East Asia as well as Australia's Northern Territory [3]. In these areas melioidosis is one of the most common causes of sepsis and severe community acquired pneumonia [4]–[7]. Infection can occur through inhalation, ingestion or percutaneous inoculation [8]. In untreated or improperly treated individuals, fatality rates from melioidosis can be as high as 95% but even with appropriate treatment melioidosis can have fatality rates up to 40% [9]. *B. mallei* is a closely-related host-adapted clone of *B. pseudomallei* [10]. It causes glanders, primarily in solipeds [11], and while endemic in parts of the Middle East, the Former Soviet Union, South and Central America [12], *B. mallei* does not persist in the environment [13]. Natural cases of glanders are rare but laboratory workers are occasionally accidentally infected [14], [15]. Like melioidosis, glanders has a 95% mortality rate in untreated individuals, which improves to only 50% in treated patients [13]. Both *B. mallei* and *B. pseudomallei* are considered potential biothreat agents and as such are ranked as Tier 1 Select Agents by the Centers for Disease Control and Prevention (CDC) [16].

Given the lethality of these bacteria, non-clinical models have proven invaluable tools in the study of host-pathogen interactions, the identification of novel virulence factors and the search for pre- and post-exposure prophylaxis against melioidosis and glanders. Large animal models including non-human primates [17] and goats [18] have recently been developed for melioidosis but are impractical for widespread use. Non-animal models such as the tomato plant (*Solanum lycopersicum*) [19] and *Caenorhabditis elegans* [20], [21], as well as insect models including wax worm larvae (*Galleria mellonella*) [22], [23] and the Madagascar hissing cockroach [24] have been employed for the identification of virulence factors of *B. pseudomallei* and *B. mallei*. However, no surrogate has been employed in the study of melioidosis and glanders to the extent of the murine model of infection. In addition to murine models being amenable to the monetary and space constraints of most investigators, mice and humans share a number of similarities, with respect to *B. mallei* and *B. pseudomallei* infection, that make mice excellent surrogate hosts for the study of melioidosis and glanders. For example, humans and mice are both susceptible to the same routes of infection with each species exhibiting multi-organ involvement primarily targeting the lungs, liver and spleen. Additionally, experimentally-infected mice produce proinflammatory cytokine signatures similar to those observed in clinical human studies [25]–[28].

A large number of inbred and outbred mouse strains have been utilized to model glanders and melioidosis[29]. However, the vast majority of studies have focused on the use of BALB/c mice to represent the acute phase of *B. mallei* and *B. pseudomallei* infection and the more resistant C57BL/6 strain to recapitulate the chronic phase of melioidosis [26], [30]–[36]. These mice can be challenged through a variety of methods and routes including oral inoculation [37], intravenous, intraperitoneal and subcutaneous injections, as well as intranasal and aerosol inhalational methods. Each of these methods produces unique results in terms of LD_50_ and disease progression and many have been thoroughly reviewed by Warawa [29]. However, because of the potential for *B. mallei* and *B. pseudomallei* to be deliberately released as an aerosolized bioweapon, much emphasis is placed on inhalational exposure routes.

Because currently accepted methods for aerosol challenges require the expert use of sophisticated equipment not available in most labs, there is a need for alternative inhalational challenge methods that are effective, inexpensive and easy to use. Here, we demonstrate oropharyngeal aspiration (OA) is an effective, inexpensive and reliable inhalational challenge method for the study of *B. pseudomallei* and *B. mallei* in BALB/c mice. Challenge doses of *B. mallei* and *B. pseudomallei* were administered by two investigators using common laboratory equipment including a pair of curved forceps and a standard pipette. The resulting disease progression is comparable to established aerosol exposure models with hematogeneous seeding of the entire host resulting in rapid morbidity and mortality typical of acute melioidosis and glanders. Finally, the interaction between pathogen and host, including pathology and host immune responses, to *B. pseudomallei* infection was assessed during this model of acute phase melioidosis.

## Materials and Methods

### Bacterial Strains and Challenge Dose Preparation

*B. pseudomallei* K96243 and *B. mallei* ATCC 23344 were obtained from BEI Resources and stored at −80°C in single use aliquots. Prior to challenge, a single aliquot was thawed, inoculated into 5 ml LB (Lennox) broth (*B. pseudomallei*) or LB supplemented with 4% glycerol (*B. mallei*) and incubated at 37°C while shaking. To ensure that the challenge dose was in the exponential phase of growth, the culture was diluted back the next morning into 25 ml of appropriate medium and returned to the shaker. The culture was grown to an OD_600_ of approximately 0.6 at which time the inoculum was diluted with PBS to obtain the desired challenge dose to be administered in 30 µl aliquots. The resulting suspensions were then serially diluted and plated on LB agar or LB + glycerol to determine the actual challenge doses administered to each experimental group.

### Animals

The experiments reported herein were approved by our Institutional Animal Care and Use Committee (protocol number AP-12-026) and conducted in compliance with the Animal Welfare Act and in accordance with the principles set forth in the “Guide for the Care and Use of Laboratory Animals,” Institute of Laboratory Animals Resources, National Research Council, National Academy Press, 1996. Naïve female BALB/c mice were obtained from the Animal Production Program at the National Cancer Institute at Frederick, MD. In all cases, mice were 6–8 weeks old at the time of arrival and were allowed one week for acclimation. Post acclimation, mice were randomly assigned to groups. The mice were maintained on a 12-hour light cycle with ad libitum access to rodent feed and water. Mice were observed a minimum of 2 times per day by the PI, associate investigator, or a member of the Vet Med staff. Early humane endpoints were determined by a combined score with a specific endpoint based on appearance (absence of grooming, piloerection, hunched), natural behavior (lack of peer interaction), and provoked behavior (unresponsive or subdued response when stimulated). Moribund animals were humanely euthanized under deep anesthesia (200 µl of ketamine [100 mg/ml] mixed with xylazine [20 mg/ml] administered ip).

### Oropharyngeal Aspiration (OA)

Oropharyngeal aspiration (OA) was utilized to challenge BALB/c mice with *B. mallei* ATCC 23344 and *B. pseudomallei* K96243. The procedure was performed on BALB/c mice that were lightly anesthetized with Ketamine [100 mg/ml] mixed with Xylazine [20 mg/ml]. Anesthesia was administered by intraperitoneal injection at a final dose of 5 µg/0.2 µg (K/X respectively). Mice were closely monitored until fully recovered and awake.

**Figure 1:**
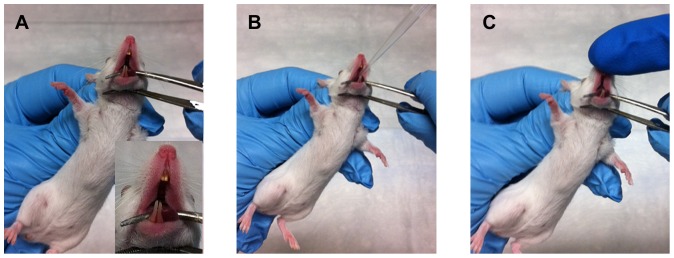
Challenge by oropharyngeal aspiration. Mice were lightly anesthetized as described in the materials and methods and manually restrained in an upright position (A). Small curved forceps were applied to gently open the mouth and secure the tongue to the lower jaw (A, inset). (B) 30 µl of inoculum was administered to the back of the mouth using a pipette and sterile tip. The nares were blocked by the second investigator (C) to prevent obligate nasal breathing and compel inhalation of the inoculums.

In this method (Fig. 1), lightly anesthetized mice were manually restrained in an upright position and curved forceps were applied to gently open the mouth and hold the tongue down to the lower jaw (Fig. 1, A - inset) to prevent swallowing. A second investigator carefully administered 30 µl of inoculum to the back of the mouth using a pipettor and sterile pipette tip (Fig. 1, B). Because mice are obligate nasal breathers, they were encouraged to aspirate the fluid into the lungs. This was accomplished by the second investigator placing a gloved finger over the mouse's nostrils (Fig. 1, C). The combination of holding the tongue to prevent swallowing and closing of the nostrils to prevent nasal breathing caused the mice to inhale through the mouth and aspirate the instilled fluid. The mice were observed to visibly inhale the inoculum and were returned to the cage to recover from anesthesia in the supine position. All mice fully recovered from the procedure within 5–10 minutes of being returned to the cage.

### 50% Lethal Dose Determination

Five groups of 15 BALB/c mice were challenged with 10-fold serial dilutions of bacteria from exponential phase cultures using the OA method described above. Challenge doses (delivered) targeting a range of 4×10^4^ cfu to 4 cfu (for Bp) or 3.8×10^5^ cfu to 36 cfu (for Bm), were administered to each group. Approximately 15 minutes following each challenge, five mice were randomly selected from each group and euthanized by exsanguination to accurately determine the inhaled dose. The lungs and trachea from the euthanized mice were aseptically harvested and homogenized in 1.0 ml of sterile PBS. The resulting suspension was serially diluted and plated to determine the actual dose inhaled by each experimental group. The remaining mice in each group (n = 10) were observed for a period of 14 days. The LD_50_ was calculated using the R statistical programming language [38].

### *B. pseudomallei* K96243 Model Development

A cohort of 50 BALB/c mice (6–8 week old females) were challenged by OA with a single dose (∼130 cfu, inhaled) of *B. pseudomallei* K96243 and evaluated every 24 hours for bacterial dissemination, histopathology and immune response. A group of 10 mice were observed to evaluate the time to death resulting from the inhaled dose. An additional control group (n = 4) was sham challenged with only PBS for histopathology and inflammatory response studies.

#### Bacterial Burden

Every 24 hours following challenge, three mice were randomly selected and euthanized by exsanguination under deep anesthesia to assess the bacterial burden in lungs, liver, spleen and blood. Blood was serially diluted in PBS immediately following harvest to prevent clotting, while the lungs, liver and spleen were weighed and homogenized in PBS. Suspensions were serially diluted and quantitatively plated in duplicate on LB agar supplemented with Kanamycin (5 µg/ml) and incubated for 72 hours at 37°C. For *B. mallei* studies, LB was supplemented with 4% glycerol and lacked antibiotic selection.

#### Bronchoalveolar Lavage (BAL)

Every 24 hours, five additional mice were randomly selected and euthanized for analysis of their lung immune response and histopathology. Bronchoalveolar lavage fluid (BALF) was collected as previously described [39]. Briefly, immediately after exsanguination, the trachea was cannulated with a SURFLO 20-gauge i.v. catheter (Terumo Medical Products, Somerset, NJ) and the lungs were gently inflated with 500 µl of PBS supplemented with 1% FBS (HyClone). The fluid was withdrawn and retained in a 1.5 ml tube. A second lavage with 750 µl of PBS +1% FBS solution was performed as before. Cells were pelleted from each sample by centrifugation (1,500×g for eight minutes) and the supernatant from the first lavage (i.e., 500 µl) was removed and stored at −80°C for cytokine analysis. The supernatant from the second lavage (i.e., 750 µl) was discarded and both pellets were resuspended to a final volume of 200 µl with PBS +1% FBS to assess inflammatory cell infiltration.

#### Histopathology

The lungs, livers and spleens harvested from the five mice euthanized at each time point (BAL, above) were fixed and maintained in 10% formalin solution (Fisher Scientific). Paraffin embedding and staining [Hematoxylin and Eosin (lung, liver and spleen) or Periodic Acid Schiff (lung)] was conducted by Histoserve Inc. (Germantown, MD) and the slides were read independently by two investigators including a board-certified veterinary pathologist. Sham challenged (PBS) lungs were harvested 72 hours following inhalation to reflect the timepoint where signs of inflammation were maximal. Lung slides, with experimental conditions concealed, were scored for signs of perivascular, peribronchial and interstitial inflammation according to previously described methods [40]–[42].

#### Immune Response Assessment

BALF was analyzed microscopically for inflammatory cell infiltration. A 10 µl aliquot from the 200 µl cell suspensions was mixed 1∶1 with Trypan Blue and the number of cells/BAL were used as a measurement of pulmonary inflammation. For serum, blood was allowed to clot at room temperature and the serum was separated by centrifugation. Serum as well as supernatant fractions from of the first lavage (500 µl) were sterilized through 0.22 µm Millipore Ultrafree-MC centrifugal filters and stored at −80°C prior to cytometric bead analysis of cytokine levels. No discernable volume was lost during filtration process. Cytokines were quantitated with a Bio-Plex 200 device using BioPlex Pro cytokine kits (BioRad) customized to quantify the cytokines IL-2, IL-4, IL-5, IL-6, IL-10, IL-12, IL 1β, TNF-α, GM-CSF and IFN-γ according to the manufacturer's instructions. BALF or serum collected from naive mice served as controls.

#### Statistics

LD_50_ calculations were performed using the R statistical programming language [38]. Cytokine expression levels, lung scores and BALF lymphocyte analysis were compared by one-way ANOVA followed by Tukey's multiple comparisons testing to discern significant differences between groups in GraphPad Prism version 5.0 (Graphpad Software, La Jolla, CA).

## Results

### Oropharyngeal aspiration is an effective method for inhalational challenge of BALB/c mice with pathogenic *Burkholderia* species

Previously, we have utilized this method to challenge A/J mice with spores of *Bacillus anthracis* 34F2 Sterne strain [43], [44]. Here, we establish oropharyngeal aspiration (OA) as an effective inhalational challenge method to deliver pathogenic *Burkholderia* species to BALB/c mice. For each pathogen, five 10-fold serial dilutions of challenge doses were administered by OA to BALB/c mice. The doses were targeted to span the previously published effective doses for each pathogen when delivered by aerosol [26], [27], [28] and ranged from approximately 10^4^ to 1 cfu for *B. pseudomallei* and 10^5^ to 10 cfu for *B. mallei*. The animals were observed for two weeks post-challenge and the resulting survival data were utilized to calculate the 50% lethal dose for each agent.

**Figure 2:**
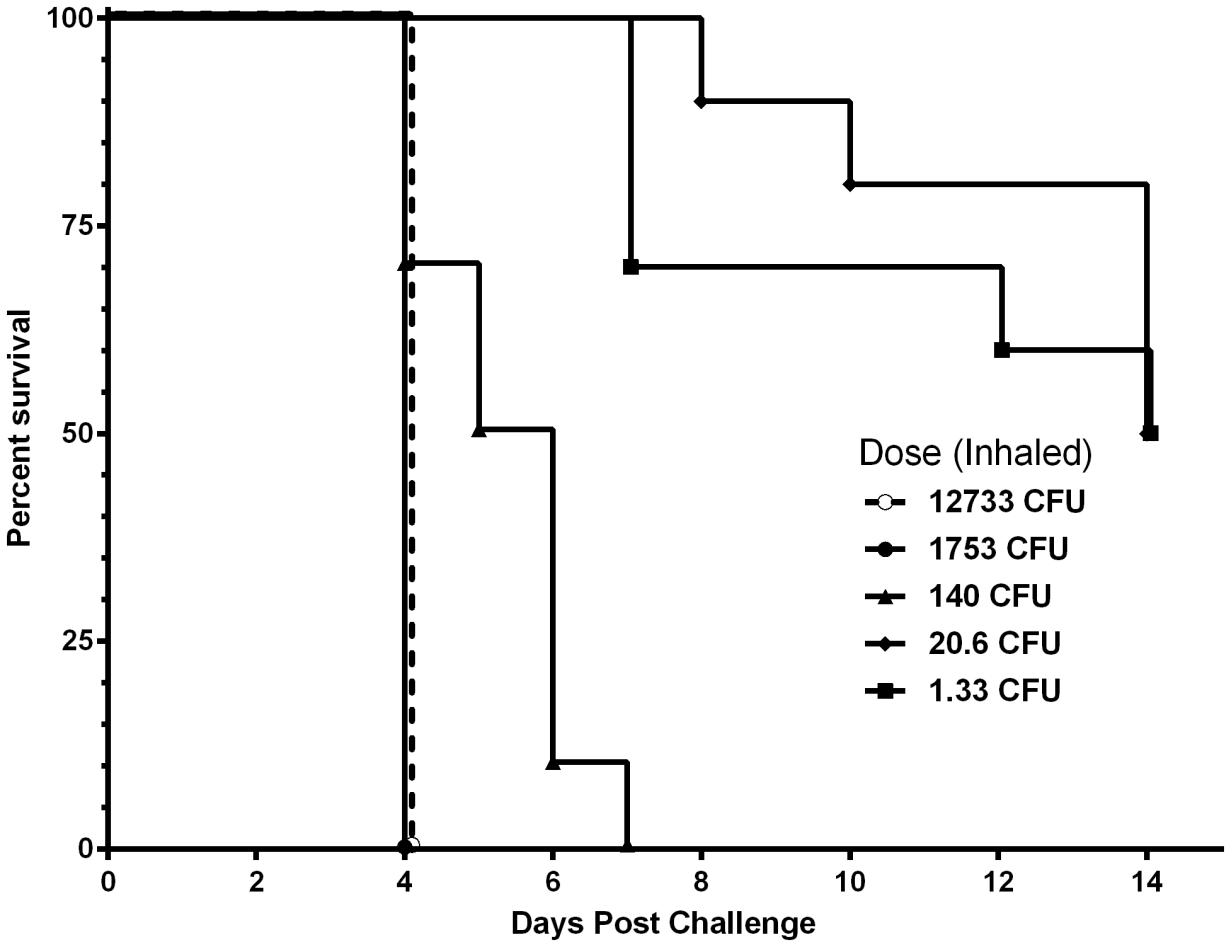
The 50% lethal dose of *B. pseudomallei* K96243 when administered by oropharyngeal aspiration. Five doses of 10-fold serial dilutions were prepared to administer 4.06×10^4^ cfu, 3.83×10^3^ cfu, 640 cfu, 40 cfu and 4 cfu of *B. pseudomallei* K96243 to BALB/c mice by OA as described as described in the materials and methods and Fig. 1. Mice were observed for 14 days. The actual inhaled doses are shown in the graph and were utilized to calculate LD_50_ (3.11 cfu).

**Figure 3:**
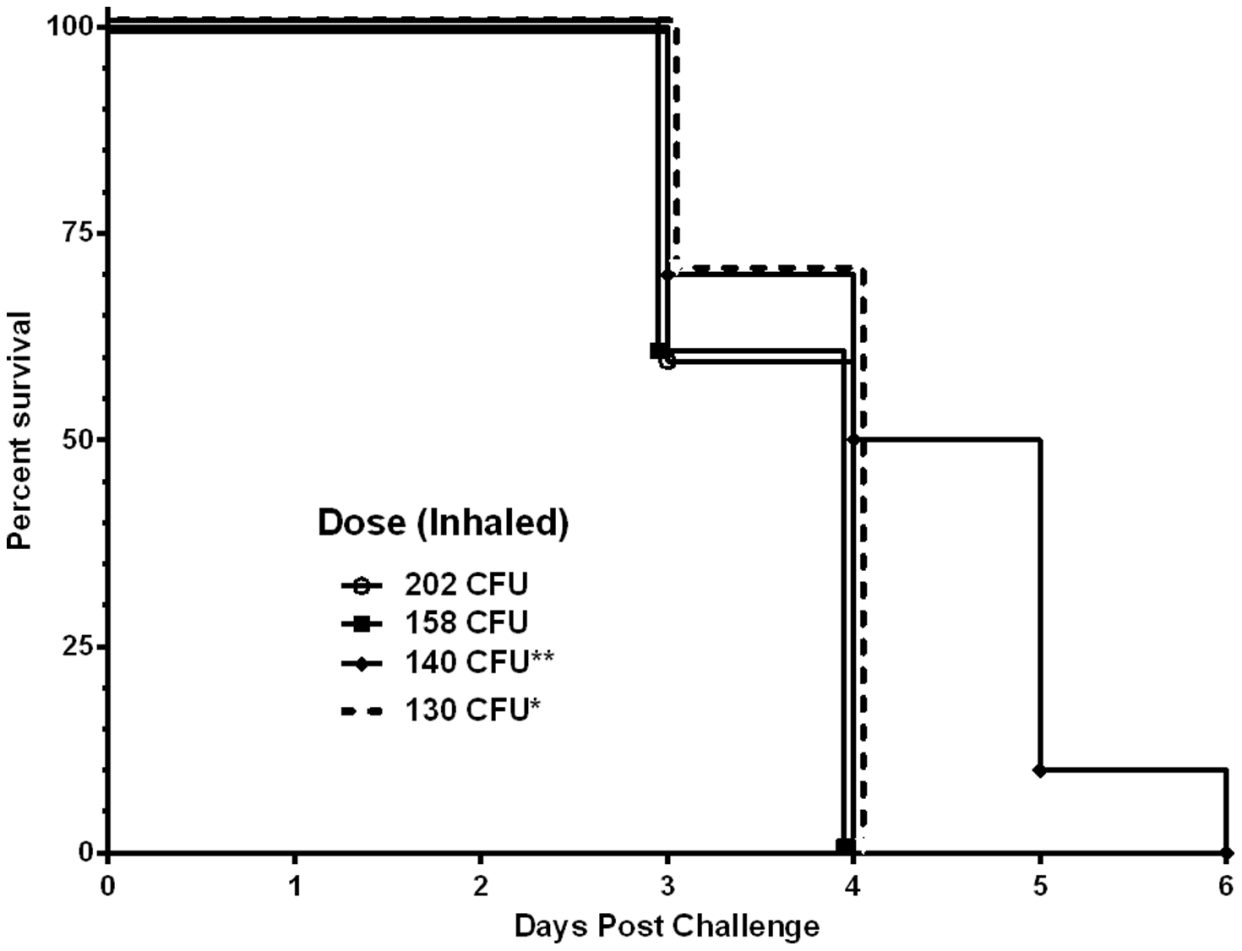
Reproducibility of oropharyngeal aspiration. The results of four independent challenges are presented here. In each case, 15 BALB/c mice were challenged by OA with *B. pseudomallei* K96243 and the inhaled dose was determed as described in the materials and methods section. Asterics identify challenges performed for this work (* from model, ** from LD_50_).

BALB/c mice are particularly sensitive to inhalational challenge with *B. pseudomallei* K96243. The reported LD_50_ for this strain, when administered by aerosol, is 10 cfu or fewer [45]–[47]. BALB/c mice were equally sensitive to this strain when it was administered by OA. Mice exhibited a dose-dependent response (Fig. 2) with the two highest doses >1,000 cfu succumbing to *B. pseudomallei* rapidly. A dose of 140 cfu was also 100% lethal but death occurred gradually with a median survival of 5.5 days. Ultimately, the 14 day LD_50_ for this strain was determined to be approximately 3 cfu when administered by OA; a result highly consistent with established aerosol challenge methods. OA was also found to be a highly reproducible method of inhalational challenge of *B. pseudomallei*. The results of four independent challenges (Fig. 3) produced nearly identical Kaplan–Meier curves.

**Figure 4:**
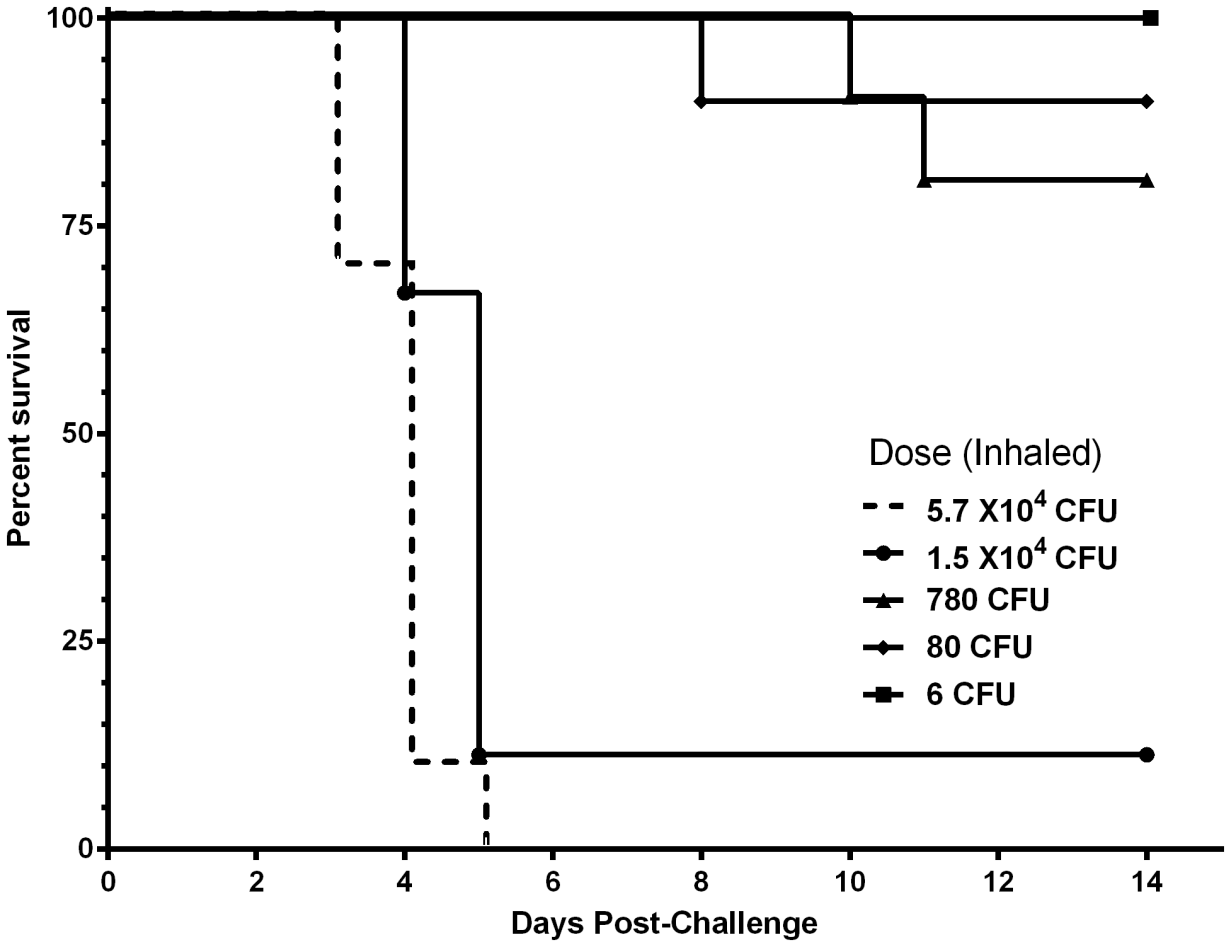
The 50% lethal dose of *B. mallei* ATCC 23344 when administered by oropharyngeal aspiration. Five doses of 10-fold serial dilutions were prepared to administer 3.8×10^5^ cfu, 3.6×10^4^ cfu, 3.36×10^3^ cfu, 366 cfu and 36 cfu of *B. mallei* ATCC 23344 to BALB/c mice by OA as described as described in the materials and methods and Fig. 1. Mice were observed for 14 days. The actual inhaled doses are shown in the graph and were utilized to calculate LD_50_ (1.7×10^3^ cfu).

This challenge method was demonstrated to be equally effective for *B. mallei* (Fig. 4) in this mouse strain. The LD_50_ for *B. mallei* ATCC 23344 when administered via OA was determined to be approximately 1.7×10^3^ CFU, nearly identical to the previously described value of 1.8×10^3^ cfu when delivered by aerosol [35].

### Oropharyngeal aspiration of *B. pseudomallei* results in systemic melioidosis

Upon the establishment of the lethality of OA delivery of *B. pseudomallei* and *B. mallei* we sought to characterize the host-pathogen interaction of *B. pseudomallei* in BALB/c mice when delivered by this method. To that end, we challenged a cohort of 50 mice with approximately 130 cfu (±7.2) of *B. pseudomallei* strain K96243 using OA. A group of ten mice were observed to characterize the lethality of the administered dose. Remaining mice were reserved for characterization of host-pathogen interactions throughout the course of acute melioidosis. Eight mice were euthanized at 24 hour intervals post infection. Three mice were randomly selected to enumerate *B. pseudomallei* colonization in the blood, lungs, liver and spleen. Bronchoalveolar lavage fluid (BALF) was collected from the five remaining mice and the lungs, livers and spleens were removed and preserved for histological analysis.

### Disease progression and host colonization

**Figure 5:**
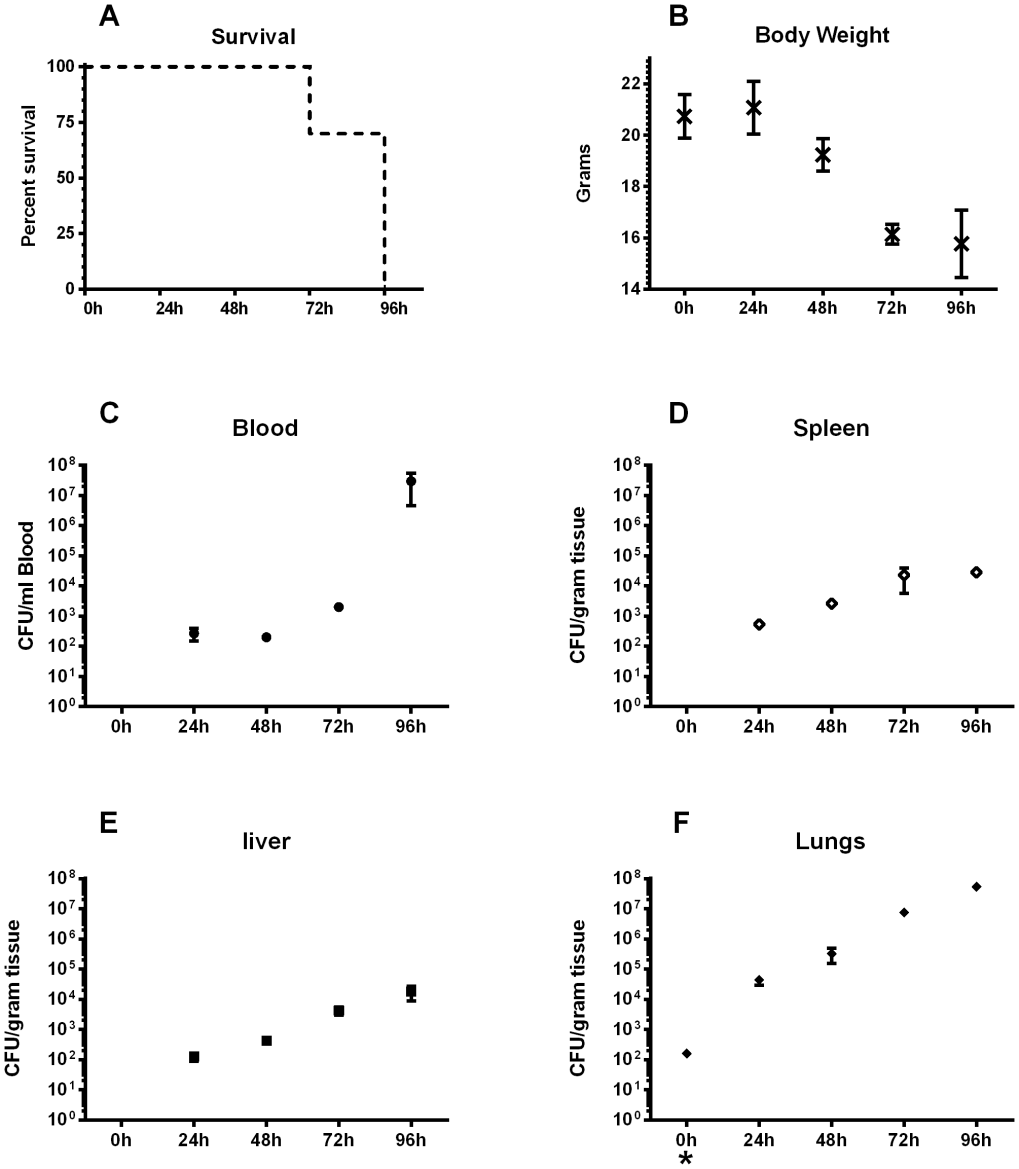
Systemic murine melioidosis resulting from oropharyngeal aspiration of *B. pseudomallei*. BALB/c mice were challenged with approximately 130 cfu (inhaled dose) of *B. pseudomallei* K96243. A group of ten mice were observed for survival analysis (A) over the duration of the experiment. At 24 hour increments, three mice were randomly selected and placed under deep Ketamine/Xylazine anesthesia. They were weighed (B), and their blood (C), spleens (D), livers (E) and lungs (F) were harvested for bacterial burden assessment. Each graph (B-F) is presented as the average cfu per gram of tissue (or ml of blood) of the three mice ± SEM. CFUs at zero hour in F (*) indicate challenge dose.

The challenge dose of 130 cfu resulted in acute murine melioidosis. Clinical signs, including rapid weight loss, conjunctivitis, loss of mobility, etc., progressed as previously described [48] and 100% mortality resulted by four days (Fig. 5, A). Challenged mice showed a rapid degeneration and lost approximately one quarter of their body weight (Fig. 5, B) over the four-day observation period relative to time zero. *B. pseudomallei* infection often presents as a bacteremia which results in hematogenous seeding of each of the host's organs [25], [30], [31], [33], [49], [50]. *B. pseudomallei* was detected in the blood as early as 24 hours post challenge (Fig. 5, C) which likely accounts for the rapid colonization of the spleen (Fig. 5, D) and liver (Fig. 5, E). The increased bacterial burden observed in the lungs (Fig. 5, F) was most likely a consequence of the challenge route. The bacteria proliferated in the lungs, liver and spleen, reaching peak levels at 96 h of 5.3×10^7^, 1.8×10^4^, and 2.8×10^4^ cfu/g, respectively, while proliferating to 3.0×10^7^ cfu/ml in the blood.

### Host temporal immune response to *B. pseudomallei*

The immune response produced in proximal and peripheral fluids was monitored temporally by collecting BALF and serum at 24 h intervals. BALF was analyzed microscopically to assess inflammatory cell infiltration into the lungs and the proinflammatory cytokine responses to *B. pseudomallei* OA challenge were analyzed at each timepoint in both BALF and serum using Luminex technology. By 96 hours, most of the infected mice succumbed to disease and the remainder met the criteria for humane endpoint euthanasia. This left only enough infected mice for bacterial burden analyses. Therefore, immune response analyses were only performed at the 24 h, 48 h and 72 h timepoints.

**Figure 6:**
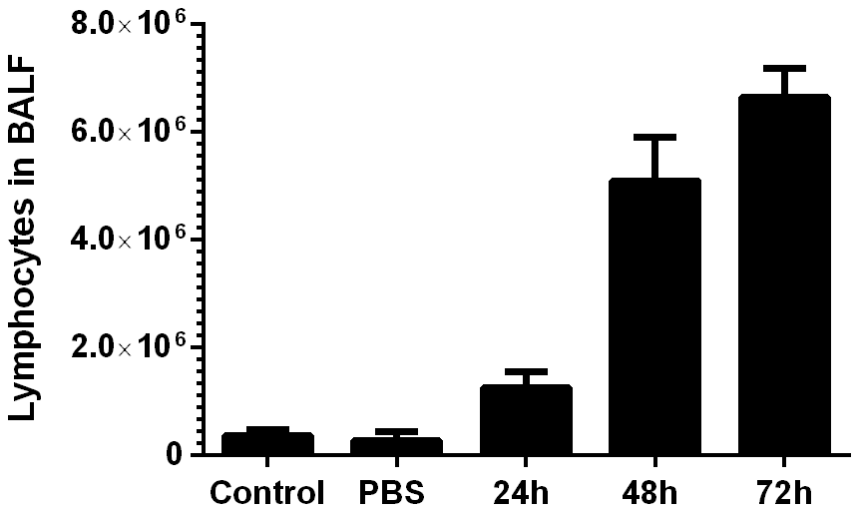
Lymphocyte infiltration of lungs following oropharyngeal aspiration of *B. pseudomallei*. At 24 hour increments, five mice were randomly selected and bronchoalveolar lavage fluid was collected. The number of lymphocytes/BALF were assessed as a measurement of pulmonary inflammation and are presented as the average of the five counts with the SEM presented on the graph. Controls depict lymphocytes from uninfected naïve mice while sham challenged (PBS) samples were harvested 72 hours following inhalation to reflect the timepoint where signs of inflammation were maximal in our studies.

Concomitant with the increase in the population of *B. pseudomallei* in the lungs (Fig. 5, F), we observed a positive correlation in the total number of inflammatory cells obtained in the in the BALF of *B. pseudomallei*-challenged mice over the control (naïve and sham challenged) BALF cell populations (*p*<0.05). These observations demonstrate an increased inflammatory response to the proliferating bacteria in the lungs and not the PBS vehicle. As observed in other lung exposure models [48], the cellular response to *B. pseudomallei* OA-challenge was characterized by neutrophil infiltration at 24 h followed by granulocyte, lymphocyte (Fig. 6), and monocyte infiltration by 48 h. Highly vacuolated or foamy macrophages were also present 48 h post-challenge (data not shown), characteristic of the types of highly-activated macrophages associated with enhanced granulomatous responses [51].

**Figure 7:**
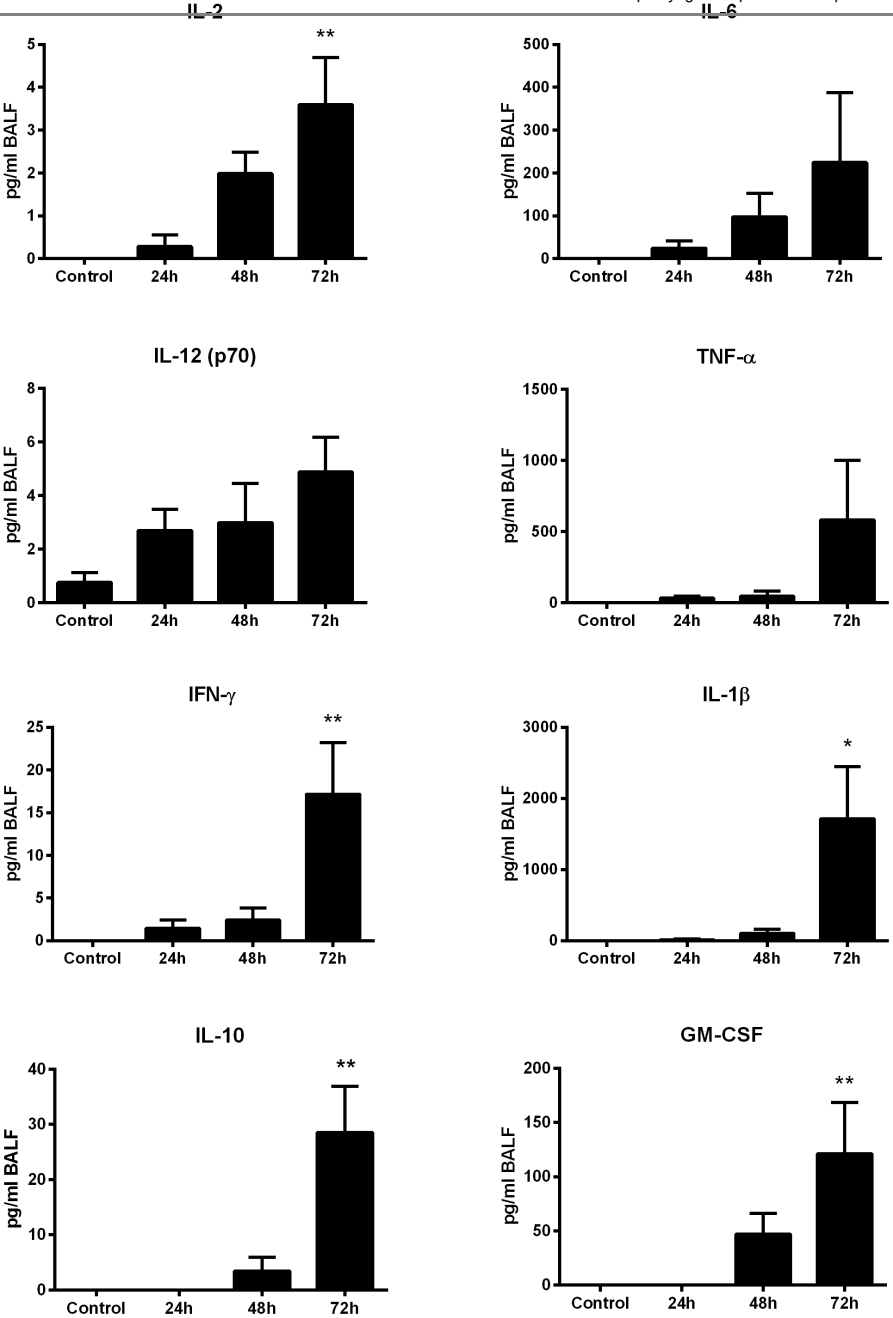
Proinflammatory cytokine production in the proximal lung fluids following oropharyngeal aspiration challenge with *B. pseudomallei*. Bronchoalveolar lavage fluid collected from OA-challenged mice (n = 5) were analyzed by cytometric bead analysis for the presence of proinflammatory cytokines. Each assay was performed in triplicate. The average of the five mice is shown in pg/ml with the SEM displayed on the graph. *indicates statistical significance compared to naïve BALF as determined by one-way Anova with Tukey's post-test (*<0.05, **<0.01).

The contribution of cell infiltration to the pulmonary immune response was assessed by Luminex-based detection of proinflammatory cytokines in BALF (Fig. 7). The levels of BALF acute phase proteins IL-6, TNF-α IL-1βand GM-CSF experienced a measurable increase by 48 h, with IL-1β and GM-CSF reaching significance by 72 hours. Similarly, measured increases the Th1-associated cytokines IL-2, IL-12, and IFN-γ were also detected in BALF 24 h post-challenge, with IL-2 and IFN-γ reaching significance by 72 h post-exposure (p<0.01). Th2-associated cytokines IL-4 and IL-5 remained below the level of detection in BALF at all timepoints, suggesting *B. pseudomallei* exposure incites a Th1-polarized response in pulmonary tissues. BALF IL-10 levels rose sharply by 72 h (p<0.01) possibly suppressing further inflammation.

**Figure 8:**
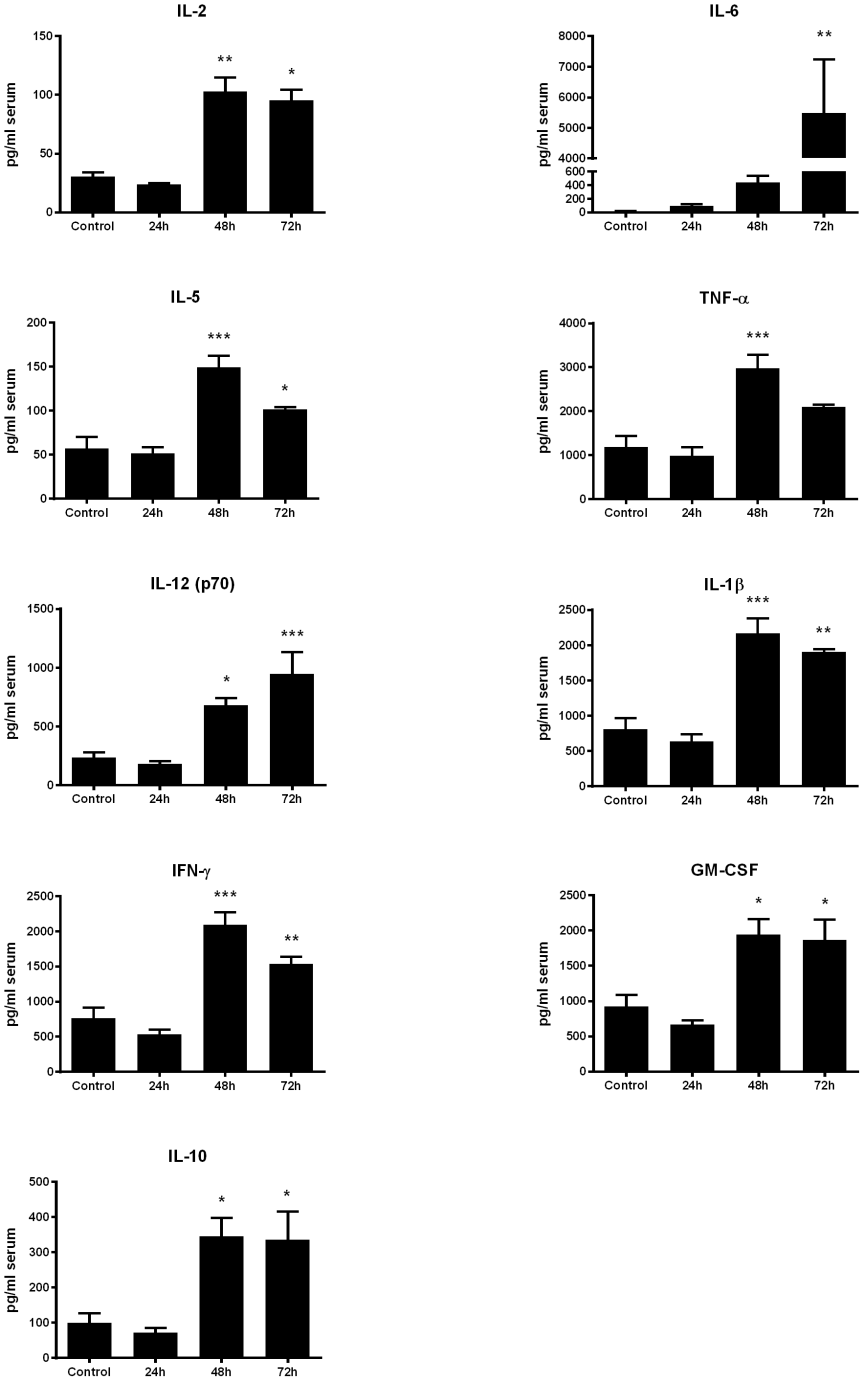
Proinflammatory cytokine concentration in the peripheral blood following oropharyngeal aspiration challenge with *B. pseudomallei*. Serum from each of the mice (n = 5) examined above was analyzed by cytometric bead analysis for the presence of proinflammatory cytokines. Each assay was performed in triplicate. The average of the five mice is shown in pg/ml with the SEM displayed on the graph. *indicates statistical significance compared to naïve serum as determined by one-way Anova with Tukey's post-test (*<0.05, **<0.01, ***<0.001).

Serum cytokine production (Fig. 8) generally resembled that observed in pulmonary tissues, although fewer cytokines were detectable in control mouse BALF than control sera. Serum levels of the acute phase proteins TNF-α IL-1βand GM-CSF peaked sharply from 24 to 48 h in infected animals, compared to naïve control mice (*p*<0.001, *p*<0.001 and *p*<0.05 respectively) followed by a partial decline by 72 h. Conversely, IL-6 did not significantly increase (*p*<0.01) compared to control animals until a sharp (12.5-fold) increase occurring between 48 h and 72 h post *B. pseudomallei* exposure. The levels of the Th1-associated cytokines IL-2, IL-12, and IFN-γ remained static relative to naïve controls at 24 h post-exposure but increased sharply by 48 h (*p*<0.01, *p*<0.05 and *p*<0.001, respectively). Serum IL-4 remained below detectable levels at each timepoint, however the serum levels of Th2-associated IL-5 and IL-10 rose sharply between 24 h and 48 h in infected animals, expressing 2.6 and 3.5-fold higher levels, respectively, than controls (*p*<0.001, *p*<0.05). Collectively, this suggests the systemic response exhibits a more balanced Th1/Th2 response than the Th1-polarized cytokine response observed in the lungs.

### Histopathological alterations in target organs

**Figure 9:**
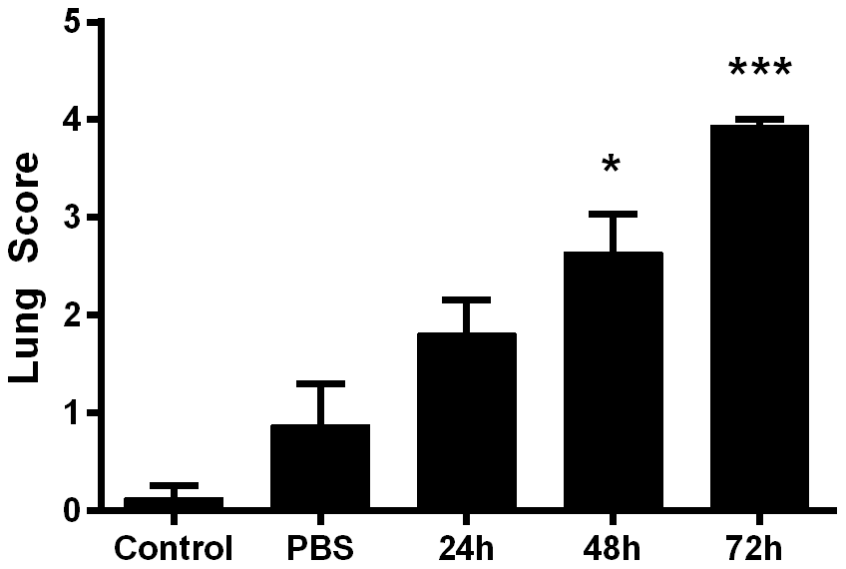
Inflammatory lung scores following OA-challenge. *B. pseudomallei* or PBS was delivered to BALB/c mice by OA. The lungs were collected and processed as described above and alalyzed for signs of perivascular, peribronchial and interstitial inflammation according to previously described methods [40]–[42]. Controls depict lungs from uninfected naïve mice while PBS represents sham challenged tissues. The average score of the five mice is shown with the SEM displayed on the graph. *indicates statistical significance compared to the PBS controls as determined by one-way Anova with Tukey's post-test (*<0.05, ***<0.0001).

**Figure 10:**
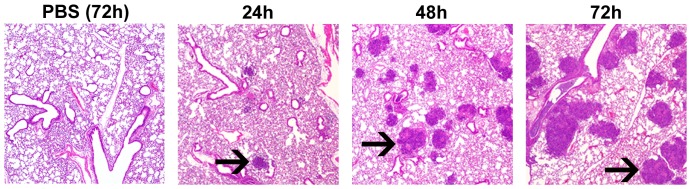
Temporal lung lesions induced by *B. pseudomallei* when administered by oropharyngeal aspiration. Lungs were collected from mice at 24 h, 48 h and 72 h post infection, stained (H&E stains and Periodic Acid Schiff) and analyzed for *B. pseudomallei* induced lung damage. Focal pyogranulomatous lesions associated with bronchioles are indicated by arrows. PBS (72 h) depict sham challenged lung tissue at 72 hours following administration of PBS. The data presented here represent a consensus of five lungs examined at each timepoint.

To examine the temporal changes in major organs targeted by *B. pseudomallei* when delivered by OA, the lungs, livers and spleens of infected mice were subjected to histopathological analysis. All tissues from infected mice showed inflammatory lesions concomitant with the increase in bacterial burden (Fig. 5). Lungs were scored for signs of perivascular, peribronchial and interstitial inflammation according to previously described methods [40]–[42]. The lungs of mice sham challenged with PBS (Fig. 9) averaged 0.875 at 72 h post administration, a score that was not significantly different than naïve mice. In contrast, this value was significantly different from the *B. pseudomallei*-infected mice at 48 h (*p*<0.05) and 72 h (*p*<0.0001). Additionally, mice sham challenged with PBS alone demonstrated no focal inflammatory lesions or any other lesions through 72 hours (Fig. 10). However, in the lungs of infected mice, pulmonary pyogranulomas initially developed around bronchioles and contained many neutrophils and macrophages with visible signs of necrosis. Lesions enlarged over the course of the experiment (indicated by→ in 24, 48, 72 hrs, Fig. 10) and were eventually located throughout the lung. By 72 hours, in all mice examined, the inflammatory foci merged into large areas of consolidation. In the liver (data not shown), we also observed the development of pyogranulomas containing neutrophils, macrophages, and necrosis. By 72 hours, thrombosis of hepatic veins and some foci of hepatocellular necrosis, associated with the thrombosis, were also present. In a similar fashion, the spleens (data not shown) of infected animals showed increasing white pulp lymphocyte apoptosis and lymphoid atrophy over time concomitant with thrombosis of veins in the red pulp.

## Discussion

Variations on the OA procedure have been extensively described in the literature for a variety of purposes including pathogen challenge [43], [44], [52], [53], toxin introduction [54], Bleomycin-induced lung fibrosis [55], induction of asthma [56], [57] and other lung damage [58]. The purpose of this study was to establish OA as a viable alternative method for inhalational challenge with *B. mallei* and *B. pseudomallei* in BALB/c mice. In these studies, OA-mediated delivery of *B. mallei* and *B. pseudomallei* (strains ATCC 23344 and K96243, respectively) yielded LD_50_ values nearly identical to those reported in previous studies administering these strains by aerosol [35], [45]–[47]. The host response to the infection was comparable previously described animal models and human clinical studies. Our data clearly define OA as a proven inhalational challenge method for *Burkholderia* pathogens in BALB/c mice.

The challenge volume of 30 µl was previously determined empirically in earlier OA studies in our laboratory using vehicle alone. During these studies, we observed demonstrable edema in the proximal lungs of BALB/c mice that inhaled greater than 30 µl of fluid (data not shown). While this edema eventually resolved over time, we utilized 30 µl as the maximal volume for studies described here to avoid potential confounding factors resulting from the inhaled fluid. The negligible effect of 30 µl of PBS, delivered by OA, on the host tissues and inflammatory response is demonstrated by figures 6, 9 and 10. As a result, all challenge doses were prepared in a per 30 µl final concentration. Additionally, 30 µl is sufficient to almost entirely fill the back of the throat of a 6–8 week old BALB/c mouse and thus is clearly visible once administered. Upon inhalation, the liquid can be clearly visualized disappearing. While it is conceivable that this method would be equally effective for other small animal challenges, the appropriate volume would have to be determined empirically for each animal model.

While this study contains no specific effort to ascertain the distribution of inoculums throughout the host, disposition in the lungs was confirmed by homogenizing the lungs in PBS and quantitatively plating on selective media. Additionally, several previous studies employing OA demonstrated preferential lung disposition using a variety of methods including labeled polystyrene latex beads [59], Evans blue dye [57] scintigraphic imaging [60] and Ventral NIRF imaging utilizing Cy5.5 [55]. In each of these cases, the material was found to be distributed throughout the lungs and not the mouth, upper airway or stomach.

In our OA model, the actual inhaled dose was often lower than the administered dose (Figures 2–4 above). In the challenges reported here, the mice inhaled approximately 33.7% (±12.6 SD) of the administered dose; however in our studies the range has varied from 10% to 35% of the administered dose. During recovery following challenge, the mice were often observed visibly and audibly coughing. We believe this action expelled some of the administered inoculum. In addition, although the lungs and lower trachea were removed for assessment of inhaled dose, the upper trachea was not. Thus, some of the administered bacteria may remain in the back of the throat and upper trachea. Finally, it is also possible that a relatively minor portion of the inoculum was swallowed, although no attempt was made to quantify the ingested amounts in this study. While we cannot definitively rule out ingestion, it is doubtful that swallowed bacteria had any impact on this study. Immobilizing the tongue (Fig. 1) prevents swallowing during the challenge procedure leaving only residual bacteria following aspiration [55], [57], [60]. Additionally, a recent study [37] demonstrated that upwards of 10^8^ *B. pseudomallei* 1026b administered enterally was required to induce acute infection. As a result, we believe that any contribution to infection from ingested bacteria was minimal.

We observed very low levels of cytokines in the BALF. The lavage process greatly dilutes pulmonary cytokines, producing measurements suitable for comparing relative, but not absolute, cytokine expression. Previous *B. pseudomallei* challenge models [61] observed Th1-polarized cytokine profiles in mouse lung homogenates consistent with the BALF Th1 profile observed in the present study, marked by the absence of IL-4 and IL-5, and temporal increases in IFN-γ, IL-12 and IL-2 expression. Protective responses against *B. pseudomallei* depend heavily upon early IL-12 and IFN-γ expression [62]. These responses act to recruit neutrophils [63] and activate macrophages [64] to produce bactericidal reactive oxygen and nitrogen intermediates, limiting the infection during its critical early stages [65]–[67].

Severe inflammation can irreversibly damage pulmonary tissues irrespective of microbial presence, producing a potentially fatal acute respiratory distress syndrome. Such damage appears in the *B. pseudomallei* OA mouse challenge model within 24 h, as indicated by the development of upper and lower airway pyogranulomas that increased overtime in number, size and degree of necrosis (Fig. 10). Damaged cells release damage-associated molecular patterns (DAMPs) that promote acute inflammation, while resolution-associated molecular patterns (RAMPs) later resolve inflammation [68]. With severe tissue damage, RAMPs accumulate to levels that induce IL-10 and other anti-inflammatory cytokines that operate in negative feedback loops to down-regulate reactive oxygen and nitrogen intermediate production [69] resolving runaway inflammation [70]. *B. pseudomallei* challenged mice exhibited a sharp increase in BALF IL-10 levels at 72 h (p<0.01) concomitant with near-peak weight loss, near-peak bacterial loads (Fig. 5), and peak organ lesion counts (Fig. 10). These observations suggest extensive damage activated inflammatory resolution and up-regulated IL-10 expression to prevent irreparable lung damage [71]. The acute phase proteins IL-6, TNF-α IL-1β and GM-CSF increased measurably by 48 h, with significant increases in IL-1βand GM-CSF by 72 h. Increases in these cytokines, particularly IL-1β, have been previously observed in mouse models *of B. pseudomallei* infection [61]. IL-1β appears to play an important role in the pulmonary inflammation associated with *B. pseudomallei* infection. For example, Ceballos-Olvera et al found that the exogenous addition of IL-1βincreased the infiltration of macrophages, neutrophils, and dendritic cells into the lungs in a mouse model of melioidosis and its production was deleterious. The converse study blocking IL-1 signaling with the addition of IL-1 receptor antagonist reduced *B. pseudomallei*-induced inflammation and pulmonary pathology [72].

Sera produced a similar cytokine profile but exhibited a more balanced Th1/2 cytokine profile as evidenced by Th2 cytokine IL-5 expression in addition to robust expression of Th1 cytokines IFN-γ, IL-12 and IL-2. In the periphery, there was also a significant increase in IL-1β which has previously been observed to correlate with an increase in c-reactive proteins (CRP) [73]. Increasing concentrations of CRP have been observed in melioidosis patients [74] and these increases have positively correlated with liver disease or dysfunction and poor clinical outcomes [75]. It is possible that the liver dysfunction often observed in melioidosis patients is contributed to by rising CRP levels mediated by IL-1β induced during *B. pseudomallei* infection. Interestingly, most serum cytokines experience a subtle decline between 48 and 72 hours with the exception of IL-6, which rose sharply in concentration. Cytokine reduction at this late time point correlated with a significant decrease in body weight and an increase in bacterial load in the blood, suggesting that these animals were becoming severely septicemic. This finding is underscored by the levels of IL-6 which did not significantly increase until 72 hours post *B. pseudomallei* exposure. Finally, high levels of IL-6 in the sera has been associated with increased bacterial loads in melioidosis patients [28], and is also associated with a negative outcome and proposed as a diagnostic marker in generalized bacterial sepsis [76]. Finally, our uninfected control mice displayed an unusually high background level of IFN-γ (∼766 pg/ml) in the serum (Fig. 8). While background levels of IFN-γ are typically near zero in naïve BALB/c mice [77], [78] the increase to ∼2,000 pg/ml was a significant increase over our baseline (<0.001) and consistent with maximal levels previously published [77].

While we did not perform an exhaustive characterization of the host-pathogen interaction for *B. mallei* when delivered by OA, we analyzed the organs of surviving mice from the *B. mallei* LD_50_ study. *B. mallei* disseminated to all of the major organs typically targeted by *B. mallei* as demonstrated in previous inhalational studies [35], [48]. Specifically, the lungs of surviving mice euthanized on day 14 were colonized with an average of 1.4×10^6^ cfu per gram of tissue, the livers contained 4.6×10^4^ cfu per gram of tissue, the spleens harbored 3.1×10^4^ cfu per gram of tissue and the blood contained 5×10^3^ cfu per milliliter of blood. Additionally, *B. mallei*–infected mice exhibited focal lesions in the lungs, liver and spleen and neutrophil-containing pyogranulomas in both the lungs and liver. Their similarities in LD_50_, bacterial dissemination, colonization of and histological damage produced in major target organs clearly define the *B. mallei* OA inhalational challenge model closely comparable to, and a viable alternative to currently-accepted aerosolized challenge models [35].

In conclusion, this study expands on current efforts to identify novel methods for inhalational challenge with biothreat agents such as *B. mallei* and *B. pseudomallei* [48], [49]. This non-invasive and technically simple method requires only standard laboratory equipment such as forceps and a pipettor, enabling as few as two researchers to execute an effective, reliable and reproducible inhalational challenge in BALB/c mice. We have further utilized this method to successfully deliver spores of *B. anthracis* 34F2 Sterne strain [43], [44] and *B. anthracis* Ames strain [unpublished studies].
